# Abattoir Wastewater Irrigation Increases the Availability of Nutrients and Influences on Plant Growth and Development

**DOI:** 10.1007/s11270-016-2947-3

**Published:** 2016-07-05

**Authors:** Raghupathi Matheyarasu, Nanthi S. Bolan, Ravi Naidu

**Affiliations:** 1 Future Industries Institute, University of South Australia, Mawson Lakes, South Australia 5095 Australia; 2 Global Centre for Environmental Remediation, The University of Newcastle, Callaghan, New South Wales 2308 Australia; 3 Cooperative Research Centre for Contamination Assessment and Remediation of the Environment, P.O. Box 486, Salisbury, South Australia 5106 Australia

**Keywords:** Abattoir, Wastewater, Irrigation, Nutrients, Phytoremediation, Plant height and dry matter

## Abstract

This study evaluated the effects of abattoir wastewater irrigation on plant growth and development. The soils used in this study were collected from Primo Smallgoods Abattoir (Port Wakefield, South Australia) at different sites such as currently irrigated (CI), currently not irrigated (CNI) and soil outside the irrigation area as control (CTRL). A completely randomised block design was employed for the plant growth experiment, where four crops *(Pennisetum purpureum*, *Medicago sativa*, *Sinapis alba* and *Helianthus annuus*) were grown separately on three different soils (CI, CNI and CTRL) in plastic pots. Two types of water (tap water and wastewater) and two loadings were applied throughout the planting period based on the field capacity (FC 100 and 150 %). The overall dry matter yield was compared between the soils and treatments. Under wastewater irrigation, among the four species grown in the CI soil, *P. purpureum* (171 g) and *H. annuus* (151 g) showed high biomass yields, followed by *S. alba* (115 g) and *M. sativa* (31 g). The plants grown under tap water showed about 70 % lower yields compared to the abattoir wastewater irrigation (AWW). Similar trends in the biomass yields were observed for CNI and CTRL soils under the two water treatments, with the biomass yields in the following order CI > CNI > CTRL soils. The results confirm the beneficial effects of AWW at the greenhouse level. However, a proper cropping pattern and wastewater irrigation management plan is essential to utilise the nutrients available in the wastewater-irrigated land treatment sites. The increase in fertility is evident from the effects of wastewater on biomass growth and also the abundance of nutrients accumulated in plants. A mass balance calculation on the applied, residual and the plant-accumulated nutrients over a few cropping periods will help us in understanding the nutrient cycling processes involved in the abattoir-irrigated land treatment sites, which will serve as an effective tool for the environmental management.

## Introduction

Wastewater reuse is an important component of sustainable water resource management; water reuse from various wastewater sources after removing the pollutants, nutrients and pathogens provides an option for water security (Grant, 2011). The management of AWW is one of the key priority areas of research to overcome Australia’s future water demand for agriculture and remediation of contaminated sites (The Australian National Water Commission, 2011). A high percentage of wastewater undergoes primary and secondary treatment before being released into the environment (Arvanitoyannis and Ladas, 2008). The discharged effluent can be used for irrigation as it is a free source of nutrients which potentially boosts production and also reduces fertiliser inputs (Toze, 2006). Abattoir wastewater is a rich source of nutrients even after primary treatment, resulting in high cost for further treatment and disposal (Li and Huang, 1986).

The abattoir wastewater (AWW) derives organic loads from different sources. For example, animal manure contributes significant amounts of pollutants to the abattoir effluent containing N, P, and organic carbon (Meat Livestock Australia (MLA), 2012). In comparison with other wastewater sources, AWW possesses the highest concentration of organic load, with high volume of COD (8000 mg/L), proteins (70 %) and suspended solids (15–30 mg/L) (Ruiz et al., 1997). Australia’s agricultural industries have experienced rapid growth in recent years, with nearly 152 abattoirs, 1798 wine industries, 9256 dairy farms and 1835 piggeries in operation. The rapid growth of Australian abattoirs has been paralleled by the number of animal slaughtered (Matheyarasu et al., 2012). The ever increasing number and volume of effluents discharged leads to a range of environmental issues in Australia such as water pollution, soil degradation and accumulation of toxic metals in plants and animals (Raghupathi et al., 2014). For instance, the production of meat results in the generation of wastewater with significant amount of pollutants, nutrients and pathogens. Moreover, these agricultural industries are also responsible for global warming and climate change (Dudgeon et al., 2006). To overcome the above problems caused by agricultural industries, sustainable alternative methods are needed which will not only reduce the pressure on global fresh water resources but also help in meeting the demands of water for households, industries, agriculture and environment (Arnell, 1999). It is most essential that industries adopt various best practices/low-cost technologies to reduce their water use and cost (Bolan et al., 2011; Bixio et al., 2006). Irrigation of wastewater is a potential low-cost approach of wastewater management and is a good source of nutrients for infertile soils (Reuter et al., 1997; Rivera et al., 1997). Australia, with several meat-based industries, needs to manage animal wastes and effluents using low-cost technologies (Australian Bureau of Statistics ABS, 2011; Australian Meat Processor Corporation AMPC, 2012). The amount of organic load, N and P and organic carbon concentration can be reduced by prior collection of manure before wash down, which will reduce effluent loading with high concentrations of pollutants (Reuter et al., 1997). Abattoir wastewater is the richest source of N and P; hence, it can be treated as an alternative source of nutrient provider for low-fertile soils (Rivera et al., 1997). Agricultural industries require additional capital to treat and discharge effluent. This is a potentially major limiting factor for the small- and medium-scale industries.

Phytoremediation of contaminated soil irrigated with effluent from agriculture industries by high biomass-producing plant species is a cost-effective technique to reduce the risk of nutrients and bioorganic compounds reaching the aquatic environment (Marmiroli et al., 2011). Using high biomass-producing plants (e.g. *Pennisetum purpureum* and *Arundo donax*) as remediators, which also have the potential to uptake high amounts of nutrients and heavy metals, can serve as a cost-effective technology (Reuter et al., 1997; Australian Meat Processor Corporation AMPC, 2012). The overall objective is to study the effect of AWW irrigation on soil fertility changes and plant productivity under greenhouse conditions. The plant growth experiment will aim to examine the effects of AWW irrigation on soil fertility and productivity of four different plant species in terms of dry matter (DM) yield without adding any other growth substances such as chemical fertilisers. Four different plant species (*P. purpureum, Medicago sativa, Sinapis alba* and *Helianthus annuus*) were grown in three soils which varied in their fertility status as affected by previous AWW irrigation. These soils include currently irrigated (CI), currently not irrigated (CNI) and control (CTRL) soils from the abattoir site at Port Wakefield, South Australia. The specific objectives of the experiments in this paper are as follows: (a) to evaluate the effects on AWW irrigation on nutrient mass balance in soil; (b) to determine the effects of AWW irrigation on plant productivity of four different species at two different irrigation rates and (c) to evaluate the most suitable plant species, irrigation intensity and irrigation type. This paper will also discuss the efficacy of high biomass-producing plant species in the high-, moderate- and low-fertile soils by altering crop irrigation and nutrient loading rates.

## Materials and Methods

### Contaminated Site Assessment and Soil Sample Collection

The study area (sampling site) is situated at 89.7 km north of Adelaide, South Australia. The latitude and longitude of the study area are 34° 8′ 26.60″ S and 138° 11′ 7.35″ E, the range is 749 m and the elevation of the treatment site is generally flat ranging from 13.5 m Australian Height Datum (AHD) to 14.5 m AHD. The region has mean annual rainfall of 287.3 mm and annual mean maximum temperature of 22.8 °C and minimum temperature of 10.7 °C.

Abattoir wastewater and soil under abattoir wastewater irrigation were collected from a land treatment site and compared with nearby control soil (CTRL). There were two treatment sites (32 ha), irrigated and non-irrigated (CI and CNI), which have been alternatively used for wastewater irrigation (with 2-year intervals). The irrigated and CTRL soil were collected, air-dried and sieved to <2 mm for physio-chemical characterisation. Both the CI and CNI sites were under long-term wastewater irrigation to manage wastewater economically, and they were used for forage production, alternatively. The land treatment site (CI) has received around 385 mm of secondary treated effluent applied over the year at the rate of 32 mm per month. The CI soil also received additional 310 mm of water through rainfall, during the period (2012). In the study site, the rate of irrigation was not adjusted according to annual rainfall, since it was intended for land treatment.

The stored soil samples as collected from different location and depths were analysed for pH, electrical conductivity (EC), nitrogen (N), phosphorus (P), carbon (C) and micro-nutrients. Soil analyses were performed following standard methods as described in the Soil chemical methods: Australasia (Rayment and Lyons, 2011) manual. Soil pH was measured in water using glass electrodes at 1:5 soil-water ratio. Soil EC was also measured at the same time using an EC metre. Soil total C and total N were estimated by dry combustion on air-dry soil using a LECO 2000 CNS analyser (Sparling et al., 2006). Olsen P was estimated by soil extraction with sodium bicarbonate (0.5 M at pH 8.5) and measured by molybdenum blue method (Olsen et al., 1954). Absorbance was measured at 882 nm in an Agilent UV–visible spectroscopy system (Germany), and the Olsen P concentration was calculated by preparing a calibration curve against the standards. The total P and micro-nutrients were determined using inductively coupled plasma-optical emission spectrometry (ICP-OES), with acid-digested soil samples (1:3 ratio of concentrated nitric-hydrochloric acid mixture/aqua regia) (Chen and Ma, 2001). Similarly, available N (nitrate-N and ammonia-N) was measured using the SKALAR SANS system (analyser) with potassium chloride (2 M)-extracted soil samples (Luo et al., 2004).

Farm nutrient budgets can be calculated using information obtained from nutrient input and plant uptake (Bennett et al., 2001), which can act as an essential tool to calculate the effective nutrient budget for avoiding nutrient loss to the environment (Oenema et al., 2003; Gourley et al., 2007). Annual nutrient loading from the slaughter house wastewater measured an average of 180 mg/L N and 30.4 mg/L P. The plant nutrient uptake can be increased when irrigated with nutrient-enriched wastewater (Morin et al., 2007). Annual nutrient loading used for the mass balance was calculated with the following equations (1 to 4):

1 $$ \mathrm{Input}\ \left(\mathrm{kg}/\mathrm{ha}\right)=\kern0.37em \frac{\mathrm{concentration}\kern0.22em \left(\mathrm{g}/{\mathrm{m}}^3\right)\kern0.37em \times \kern0.37em \mathrm{Irrigation}\ \mathrm{load}\kern0.37em \left({\mathrm{m}}^3/\mathrm{ha}\right)}{1000} $$

2 $$ \mathrm{Uptake}\kern0.37em \left(\mathrm{kg}/\mathrm{ha}\right)=\kern0.37em \mathrm{Dry}\ \mathrm{mater}\ \mathrm{yield}\ \left(\mathrm{kg}/\mathrm{ha}\right)\times \mathrm{Nutrient}\ \mathrm{concentration}\ \left(\mathrm{g}/\mathrm{kgDM}\right) $$

3 $$ \mathrm{Soil}\ \mathrm{surplus}\ \mathrm{estimated}\kern0.37em \left(\mathrm{kg}/\mathrm{ha}\right)=\kern0.37em \mathrm{input}\left(\mathrm{kg}/\mathrm{ha}\right)-\mathrm{plant}\ \mathrm{uptake}\left(\mathrm{kg}/\mathrm{ha}\right) $$

4 $$ \mathrm{Soil}\ \mathrm{surplus}\ \mathrm{measured}\kern0.37em \left(\mathrm{kg}/\mathrm{ha}\right)=\kern0.37em \frac{\mathrm{m}\mathrm{easured}\ \mathrm{concentration}\left(\mathrm{m}\mathrm{g}/\mathrm{kg}\ \mathrm{of}\ \mathrm{soil}\ \right)\kern0.5em \times \kern0.5em \mathrm{B}\mathrm{D}\ \left(\mathrm{kg}/{\mathrm{m}}^3\right)\kern0.5em \times \kern0.5em \mathrm{depth}\kern0.37em \left(\mathrm{m}\right)\kern0.37em \times \kern0.5em \mathrm{area}\kern0.5em {10}^4{\mathrm{m}}^2/\mathrm{ha}}{10^6} $$

### Plant Growth Experiment

The plant growth experiment was conducted at the University of South Australia greenhouse using the contaminated soil collected from the land treatment sites. The wastewater used in this experiment was collected from the Primo abattoir at Port Wakefield, which was rich in major plant nutrients such as total nitrogen (TN) and total phosphorus (TP).

**Plate 1:**
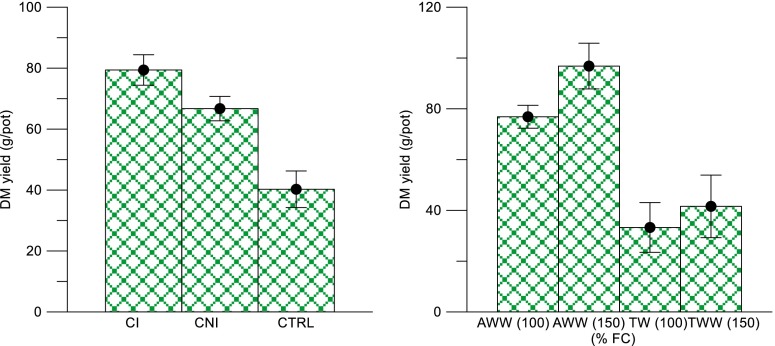
**a**, **b**, **c**, **d** Testing the biomass productivity of bioenergy crops under greenhouse condition

A completely randomised block design was employed for the plant growth experiment, where four plant species (*P. purpureum*, *M. sativa*, *S. alba* and *H. annuus*) were grown separately on three different soils (CI, CNI and CTRL) in plastic pots (W177 mm, H175 mm, L 177 mm). The pots were irrigated with AWW and TW at two different loading rates (100 and 150 % of FC; based on current practice), and each treatment was replicated three times. The objective for using two different nutrient loading rates was to study the effects of AWW irrigation on soil fertility, plant growth and total dry matter yield (DM yield). The plants were harvested 120 days after planting, oven-dried and used for analyses (Plate 1).

**Fig. 1:**
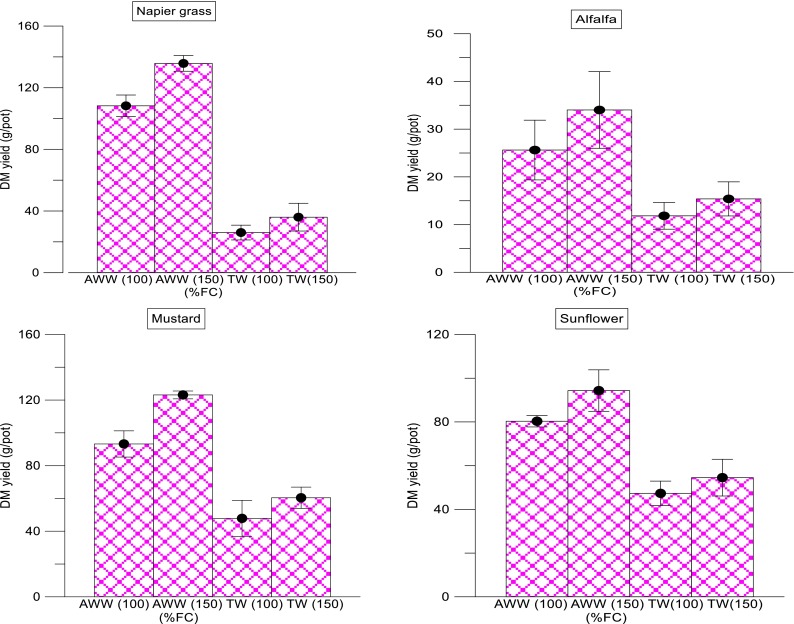
Effects of soil and nutrient loading types on the total dry matter production (*Error bars* represent the standard deviation between the replicates) (*CI* currently irrigated, *CNI* currently not irrigated, *CTRL* control, *AWW* abattoir wastewater; *TW* tap water, *FC* field capacity, *NG* Napier grass, *ALF* alfalfa, *MUS* mustard, *SF* sunflower

### Characterisation of Plant and Soil Samples After Harvest

After harvesting the plants, the roots and shoots were separated, washed with Milli-Q water and the fresh weight was recorded. The samples were then dried to constant weight at 70 °C by using a forced-air oven and ground to a fine powder for TN and TP analysis. The ground plant material (0.4 g) was weighed directly into a 75-mL digestion tube. Approximately 5 mL of concentrated nitric acid was added and left to cold digest in a fume cupboard overnight. After keeping the samples overnight, the tubes were heated using a temperature-controlled digestion block (AI Scientific Block Digestion System AIM 500) programmed to slowly increase the temperature to 140 °C until approximately 1 mL of the digest remained in the tube. The tubes were brought to room temperature prior to dilution with Milli-Q water. The samples were mixed thoroughly and filtered with a syringe filter directly into plastic containers. The digested plant extracts (along with blanks) were analysed for TP by ICP-OES (Agilent). After the destructive harvest of plant material, the soil was carefully removed from the pot and dried and stored for further physicochemical analyses. The soil samples were analysed for TN, nitrate-N, ammonia-N, TP and Olsen P after the experiment.

### Statistical Analysis

SPSS (Inc., 2001) was used to analyse the data (Pearson correlation and two-sample *t* test). The differences in the replicates were determined using standard deviation for the chemical properties of materials used and also the experimental results. Relationships between AWW and plant growth parameters were analysed using a Pearson correlation coefficient. The difference between the AWW and TW irrigation was studied using a two-sample *t* test and the significant level was *p* < 0.001.

## Results and Discussion

### Properties of Soils and Wastewater Used in this Experiment

The soils used in this study were collected from an abattoir wastewater discharged landfill site at different points such as currently irrigated (CI), currently not irrigated (CNI) and soil outside the irrigation area as control (CTRL). The collected samples were air-dried, characterised for physicochemical properties and used for greenhouse plant growth experiment. Soil collected from the land treatment site was moderately alkaline; the pH of the CI soil was moderately acidic (6.3), with CNI and CTRL measuring 8 and 8.6, respectively. The electrical conductivity was very high ranging from 500 to 1109 μS/cm. The nutrient contents in CI were up to 2080 mg/kg of nitrogen (N) and 489 mg/kg of phosphorus (P); in the CNI soil, it was 1634.6 mg/kg of N and 327 mg/kg of P; and in the control soil, 669 mg/kg of N and 98.6 mg/kg of P were recorded. The CNI soil also expressed a high concentration of major nutrients but comparatively lower than the CI and significantly higher than the CTRL soil. The wastewater sample was characterised for their major nutrient concentration (N and P) immediately after collection. The CI soil was high in TN and TP at available nutrient concentrations. The AWW used in this experiment contained high TN and TP concentration (186 mg/L of N and 30.4 mg/L of P). Generally, wastewater irrigation promote plant growth directly by either facilitating resource acquisition (nitrogen, phosphorus and micronutrients) or modulating plant hormone levels or indirectly by decreasing the inhibitory effects of various pathogens on plant growth and development in the forms of biocontrol agents (Glick, 2012). The AWW irrigation had significant impacts on the soil physiochemical property of the soils.

The soil irrigated with abattoir wastewater had a significant increase in the nutrient content of both macronutrients (N, P, K) and micronutrients (Ca, Mg, Zn, Fe, Al, Bo) compared to the non-irrigated control samples. Long-term wastewater irrigation (>22 years) can cause changes in soil properties, especially high loading of C, N and P; due to this, microbial C were doubled (Sparling et al., 2000). The soil characterisation (postharvest) results showed that there is a considerable variation in the primary nutrient (N and P) concentration between the samples of CI, CNI and CTRL. For example, the TN concentration of CI soils increased about 19 % as compared to control. Similarly, TP increased to about 253 % in CI as compared to control pots. The similar response was found in the CNI soil as well. In these experimental pots, the TP content increased to about 179 % in CNI soil pots compared to control. On the other hand, TN decreases up to 10 % as compared to CTRL soil, which was never been irrigated with nutrient-enriched AWW. The increases in soil nutrient concentration can enhance the DM yield on the above ground (Truu et al., 2009). The total N content of the CI soil (1764.3 mg/kg) was significantly higher than the CTRL soil (1483.1 mg/kg). Similarly, TP followed a similar pattern, which increased from 28.7 mg/kg (CTRL soil) to 223 mg/kg (CI soil) (Table 1).

**Table 1:** Effects of abattoir wastewater irrigation on soil properties and plant growth and development (mean ± s.d.; *n* = 114; comparison of the effects of three types of soils = CI, CNI and CTRL soils)

Properties	CTRL	CI	Sig. diff	CTRL	CNI	Sig. diff.	CI	CNI	Sig. diff.
Total N (mg/kg)	1483.1 ± 12.3	1764.3 ± 14.3	n.s	1483.1 ± 12.4	1337.9 ± 9.7	n.s	1764.3 ± 143	1337.9 ± 90.7	n.s
Nitrate-N (mg/kg)	91.5 ± 0.9	164.5 ± 1.4	*p* < 0.001	91.5 ± 0.9	118.9 ± 1.4	n.s	164.5 ± 11.4	118.9 ± 13.4	n.s
Ammonia-N (mg/kg)	18.7 ± 0.1	56.9 ± 0.5	*p* < 0.001	18.7 ± 0.1	23.9 ± 0.3	*p* < 0.001	56.9 ± 5.2	23.9 ± 3.3	*p* < 0.001
Total P (mg/kg)	94.2 ± 1.8	332.7 ± 3.4	*p* < 0.001	94.2 ± 1.8	263.2 ± 1.3	*p* < 0.05	332.7 ± 34	263.2 ± 13.6	n.s
Olsen P (mg/kg)	36.3 ± 0.3	123.3 ± 1.4	*p* < 0.001	36.3 ± 0.3	67.7 ± 0.8	*p* < 0.001	123.3 ± 14.7	67.7 ± 8.2	*p* < 0.001
K (mg/kg)	331 ± 5	5512 ± 23.4	*p* < 0.001	331 ± 5.3	6487.7 ± 17.5	*p* < 0.001	5512 ± 234.5	6487.7 ± 172.5	*p* < 0.001
Tillers	2.8 ± 0.4	5.1 ± 0.1	n.s	2.8 ± 0.4	4.2 ± 0.6	n.s	5.1 ± 1	4.2 ± 0.6	n.s
DM yield (g)	40.2 ± 0.3	79.3 ± 0.7	*p* < 0.001	40.2 ± 0.7	66.7 ± 0.6.	*p* < 0.001	79.3 ± 7.4	66.7 ± 6.2	n.s

*ns* not significant

### Effect of Different Soil Types on Plant DM Yield in Four Different Crops Irrigated with Wastewater

The effect of AWW irrigation on soil fertility and plant biomass yield of the selected plant species (*P. purpureum*, *M. sativa*, *S. alba* and *H. annuus*) were examined. The results showed that the application of AWW to the low-fertile soils resulted in an increase in soil fertility status. The total DM yield significantly increased in both CI and CNI pots as compared to the CTRL; however, the yield increase in CI versus CNI was moderate (Fig. 1). In CI soil, the overall dry matter production was higher (80 g/pot) compared to that of the CNI (66 g/pot) and CTRL soil being 40 g/pot based on a combination of all treatments. The DM production reflected the soil nutrient contents with mainly TN and TP present in the soils. Sparling et al. (2001) observed increases in total DM production in CI soils. Similarly, Picchioni et al. (2012) found that CI soil produced higher biomass yield than CNI did. Both authors concluded that these changes in biomass production were caused by the nutrient addition through effluent irrigation.

The difference in the total DM production of the three different soils may be attributed to the difference in the amount of total and available nutrients in the soil such as nitrate-N and Olsen P. There was a statistically significant effect of AWW irrigation on soil available nutrients in the CI and CNI treatments (Table 1). AWW irrigation and different loading rates had significant impacts on the soil properties. In comparison with TW, AWW showed significant increases in the soil nutrient content. In the current pot experiment, the soil irrigated with AWW had a significant increase in the nutrient contents of both total (TN and TP) and available (nitrate-N, ammonia N, Olsen P) compared to the TW-irrigated pots. The soil characterisation results after plant harvest showed that there was a considerable variation in the primary nutrient concentration and available nutrients between the samples of CI, CNI and CTRL and the extended treatments of AWW 100 % FC and AWW 150 % FC (Fig. 1). For example, the soil types (CI and CTRL) were compared for all AWW treatments and most of the nutrients (TN, nitrate-N, ammonia-N, TP, Olsen P) significantly increased (*P* < 0.001) in the same treatment, and total DM production was also significantly different (*P* < 0.001) (Table 2). Wastewater irrigation can enhance soil fertility and productivity of the soil through increasing levels of plant nutrients and soil organic matter (Mohammad and Mazahreh, 2003), and they concluded that secondary treated wastewater can improve soil fertility parameters. However, proper irrigation management and periodic monitoring of soil quality parameters are required to minimise the adverse effects on the soil. Based on the data presented by Jiménez (2005), the addition of nitrogen and phosphorus increased the productivity and phosphorus accumulation in soils increased phosphorus absorption by plants. Wastewater irrigation in the Tula Valley in Mexico provides 2400 kg of organic matter, 195 kg of nitrogen, and 81 kg of phosphorus ha^−1^ year^−1^, contributing to significant increases in crop yields (Jiménez, 2005). Vazquez-Montiel et al. (1996) observed that the main removal mechanism for N during irrigation was crop uptake whereas P was removed primarily by soil processes. The fertiliser value of the treated effluent was demonstrated by increased crop yields, and the N yield was within the range of expected values for this crop.

**Table 2:** Effects of abattoir wastewater irrigation on soil properties and plant growth and development (mean ± s.d.; *n* = 114; comparison of overall effects of four treatments (AWW and TW at 100 and 150 % FC each) against three types of soils (CI, CNI and CTRL soils)

Properties	AWW 100 FC	TW 100 FC	Sig. diff.	AWW 150 FC	TW 150 FC	Sig. diff.
Total N (mg/kg)	1578.6 ± 12.1	1130.1 ± 9.2	*p* < 0.005	2363.2 ± 14.3	1041.9 ± 8.7	*p* < 0.001
Nitrate-N (mg/kg)	140.2 ± 1.3	69.3 ± 0.9	*p* < 0.001	197.1 ± 1.2	93.5 ± 0.6	*p* < 0.001
Ammonia- N (mg/kg)	35 ± 0.3	27.5 ± 0.4	n.s	41.9 ± 0.5	28.2 ± 0.3	n.s
Total P (mg/kg)	247.3 ± 2.9	158.2 ± 2.4	n.s	347 ± 3.4	167.7 ± 2.9	*p* < 0.001
Olsen P (mg/kg)	90.8 ± 1.2	36.3 ± 0.5	*p* < 0.001	130 ± 1.7	46.2 ± 0.7	*p* < 0.001
K (mg/kg)	3730.1 ± 43.5	4459.2 ± 54.1	n.s	4035.1 ± 46.2	4216 ± 46.2	n.s
Tillers	5 ± 1	2.4 ± 0.3	n.s	5.6 ± 1	3.2 ± 0.5	n.s
DM yield (g)	76.9 ± 0.7	33.3 ± 0.6	*p* < 0.001	96.8 ± 0.8	41.5 ± 0.8	*p* < 0.001
Plant height (cm)	88.9 ± 6	91.2 ± 5	n.s	92.2 ± 6	91.6 ± 4	n.s

*ns* not significant

### Effect of Different Loading Rates on Plant DM Yield of Four Different Crops Irrigated with Wastewater

**Fig. 2:**
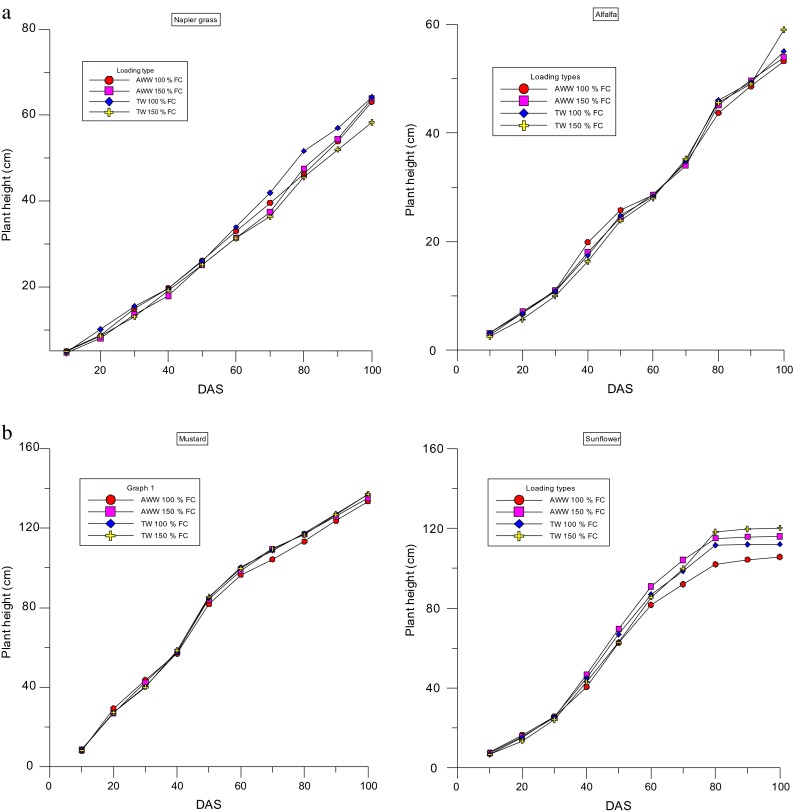
Effects of irrigation rates of abattoir wastewater and tap water on the dry matter yield of each crop grown in wastewater-irrigated soils. *Error bars* represent the standard deviation (*NG* Napier grass, *ALF* alfalfa, *MUS* mustard, *SF* sunflower)

The DM yield data obtained from the plant growth experiment of all the four crops (*P. purpureum*, *M. sativa*, *S. alba* and *H. annuus*) showed similar trends of increased yield under highly fertile conditions. The CI soil irrigated with AWW (100 and 150 % FC), showed higher yields compared to TW irrigation under the same loading rates (Fig. 2). The CNI and CTRL soils showed similar trends. This is attributed to the supply of high rate of irrigation and the resultant nutrients supplied; wastewater supplies readily available plant nutrients such as nitrate-N, ammonia-N and Olsen P for the better growth and development of the plants. This resulted in increased plant growth and development thereby effecting high DM production. Picchioni et al. (2012) used secondary treated industry wastewater with various irrigation flow systems (effluent alone and effluent plus rainfall) to study their effect on soil fertility and DM yield of natural vegetation of Chihuahuan desert shrubland (*Larrea tridentata*, Coville and *Prosopis glandulosa* Torr. var. *glandulosa*). They observed that 78 % of the increase in dry matter production in the irrigated soils was due to effluent addition which supplied sufficient nutrients (Picchioni et al., 2012). Similarly, a study by Ercoli et al. (1999) found that irrigation did not modify the biomass yield without sufficient nutrient supply; increased irrigation rate with increased N supply increased the DM yield to 37 t ha^−1^ year^−1^ with N supply and irrigation. To substantiate the above observation, similar results were found in this research; the combination of high rate of irrigation (AWW 150 %) with sufficient nutrient concentration resulted in higher DM yield compared to lower irrigation rates with poor nutrient supply (TW 100 % FC). In the case of CTRL soil, the overall yield showed less influence of TW irrigation compared to AWW, whereas there was an increase in DM yield on the AWW-irrigated CTRL pots. There were no significant differences in the plant DM yields between the TW 100 % and TW 150 % in the CTRL soil in the current plant growth experiments. In the case of crops, AWW irrigation showed significantly higher biomass yields compared to TW as evident from AWW’s nutrient richness (Figs. 2). In *P. purpureum*, at the highest treatment (150 % FC), AWW irrigation produced 275 % higher biomass yield compared to TW at the same rate.

The yields for *M. sativa*, *S. alba* and *H. annuus* under AWW irrigation were 126, 183 and 74 %, respectively, compared to TW irrigation. However, in the case of loading rates of AWW and TW individually, only *P. purpureum* and *S. alba* showed significantly higher biomass yield (Fig. 2). There was no significant effect of two loading rates in TW, which suggested that moisture content was sufficient at 100 % FC and that the nutrients were responsible for the difference in yields. A Pearson correlation matrix was done for all the types of wastewaters and the two loading rates used in this study. The effects of the major and micronutrients in the soil were positively correlated with the soil TN and TP. For example, nitrate-N, TP, Olsen P, K and shoot diameter were positively correlated with soil TN concentration. Similarly, the total number of tillers, shoot diameter and plant height were positively correlated with TP. On the other hand, the following correlation matrix was observed under the AWW treatment (alone). The TN was positively correlated with the TN, TP, Olsen P and DM yield. In the same treatment condition, Olsen P was positively correlated with the total number of tillers and DM yields of the plant species used. Similarly, in the TW (alone) treatment, the TN was positively correlated with TP and shoot diameter. Under the same treatment conditions, Olsen P was negatively correlated with the plant height of the plants species used (Table 3). Wastewater is reused for agriculture is efficient and safe, considering both farmers’ needs and health protection measures (Rosenqvist et al., 1997). According to Rosenqvist et al. (1997), an increased wastewater application rate, increased biomass production and the reduced costs for the farmer have a limited impact on the economical result. However, an increased biomass production probably enables an increased N-application without risks for N-leakage. Based on a study by Rusan et al. (2007), long-term wastewater irrigation increased salts, organic matter and plant nutrients in the soil. Also, they concluded that the biomass increased with added wastewater and nutrients provided with the wastewater. However, a longer period of wastewater application (10 years) resulted in lower biomass production but remained higher than that of the control plants. Plant essential nutrients (Total-N, NO_3_^−^, P, and K) were higher in plants grown in soils irrigated with wastewater (Rusan et al., 2007).

**Table 3:** Pearson correlation coefficients of AWW- and TW-irrigated soil properties and plant growth parameters (*n* = 114) (overall effects on three types of soils = CI, CNI and CTRL soils)

Properties	Total N (mg/kg)	Nitrate-N (mg/kg)	Ammonia-N (mg/kg)	Total P (mg/kg)	Olsen P (mg/kg)	K (mg/kg)	Tillers	DM yield (g)	Plant height (cm)
Total N (mg/kg)	1								
Nitrate-N (mg/kg)	0.007	1							
Ammonia-N (mg/kg)	0.430^b^	0.637^b^	1						
Total P (mg/kg)	0.688^b^	−0.004	0.248	1					
Olsen P (mg/kg)	0.443^b^	0.187	0.298	0.578^b^	1				
K (mg/kg)	0.411^a^	0.238	0.255	0.749^b^	0.331^a^	1			
Tillers	0.319	−0.331^a^	0.055	0.608^b^	0.282	0.312	1		
DM yield (g)	−0.420^a^	0.564^b^	0.174	−0.125	−0.039	0.301	−0.292	1	
Plant height (cm)	−0.492^b^	0.32	−0.121	−0.404^a^	−0.265	−0.052	−0.690^b^	0.733^b^	1

^a^Correlation is significant at the 0.05 level

^b^Correlation is significant at the 0.01 level

### Effects of Wastewater Types and Loading Rate on the Plant Height of the Four Plant Species

**Fig. 3:**
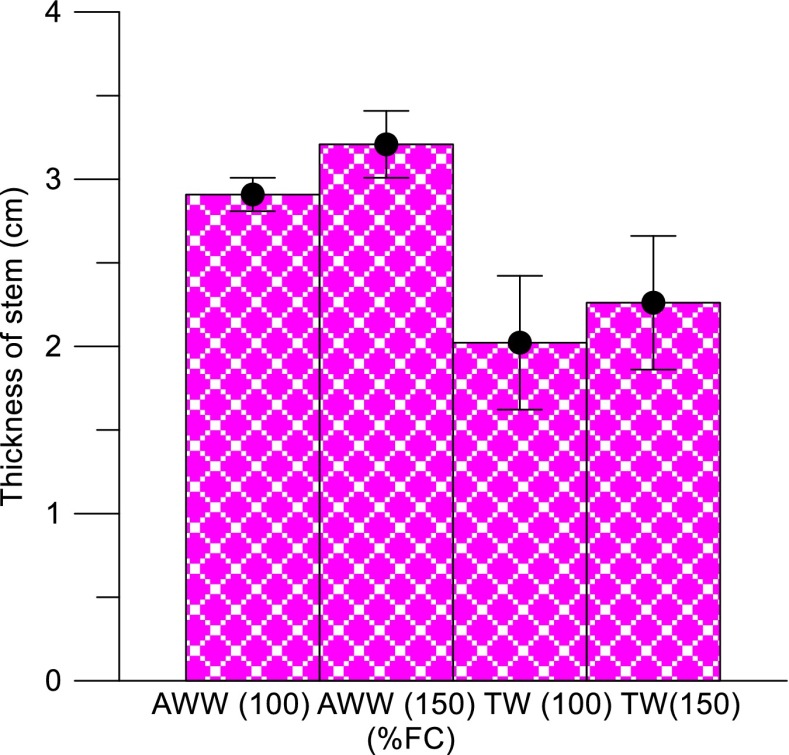
**a** Effects of nutrient loading types on plant height of Napier grass and alfalfa, (*AWW* abattoir wastewater, *TW* tap water, *FC* field capacity). **b** Effects of nutrient loading types on plant height of mustard and sunflower (*AWW* abattoir wastewater, *TW* tap water, *FC* field capacity)

Over the study period, changes in plant growth were measured every 10 days by recording the plant height. The measurement was taken to study the changes between the wastewater treatment (AWW vs TW) and differences between the loading rates (100 and 150 % FC). Figure 3 shows that there were no significant differences between the AWW 100 and AWW 150 % FC in the plant height. During the first phase of the growth (0–30 DAS), there was a minimal difference in growth (in terms of plant height) and at the middle stage (60–90 DAS), there were no significant differences and at the end of the cropping cycle, there was little difference in the plant height of the various treatments used (Plates 1). There were not many differences in the plant height of the four crops tested with four different combinations (nutrient loading pattern). The height of the plant at each treatment which was measured at 10-day intervals is presented in Fig. 3a, b, and the maximum height of the four crops was in the following order: *P. purpureum* reached approximately 63 cm; *M. sativa* 59 cm, *S. alba* 136 cm and *H. annuus* reached a maximum of 120 cm at the time of harvesting (Fig. 3).

### Effects of Wastewater Types and Loading Rate on the Plant Thickness of Stem of the Four Plant Species

**Fig. 4:**
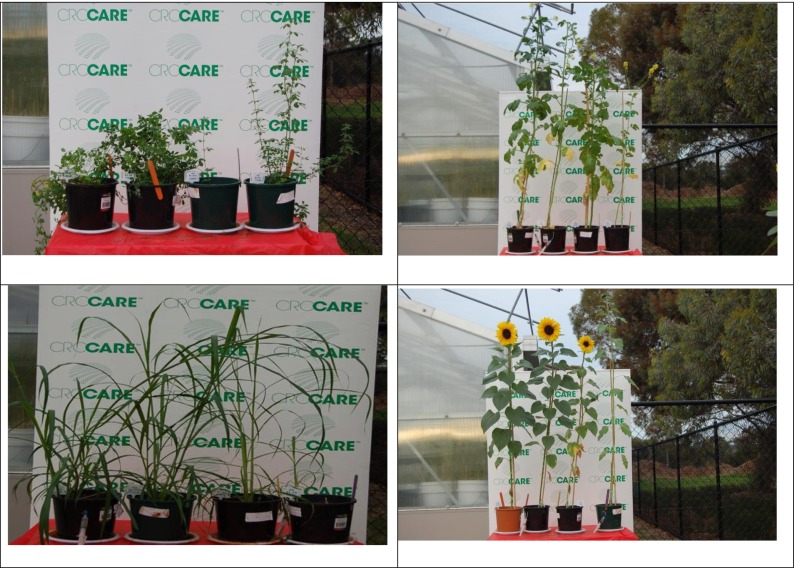
Effects of water types and nutrient loading on the thickness of stem of the four different plant species. *Error bars* represent the standard deviation (*AWW* abattoir wastewater, *TW* tap water, *FC* field capacity)

At the end of the plant growth experiment, thickness of stem was measured. The changes in stem thickness were measured in 100 DAS, at the time of harvesting. The measurement was taken to study the changes between the wastewater treatment (AWW vs TW) and differences between the loading rates (100 and 150 % FC). There was a significant difference between wastewater and TW irrigation; the wastewater-irrigated pots showed an average stem thickness of 3 to 3.2 cm, whereas in TW-irrigated pots, the thickness was only 2–2.2 cm. Figure 4 shows significant differences in the thickness of stem between the AWW (100 and 150 %) and TW (100 and 150 %) treatments. Dairy effluent irrigation can increase plant growth and produce nutritious biomass (percentage of biomass as leaves) thus resulting in increased nutrient cycling (Marmiroli et al., 2012). Stewart et al. (1990) found that land disposal of municipal effluent by irrigating tree crops is feasible on the high rates of wood production.

Based on a research by Luo et al. (2004), it is concluded that application of meat processing effluents can increase plant production due to increased nutrient loadings. According to this research, about 23–45 % of the applied effluent N and 22–31 % of the applied effluent P were recovered in the plants with increased annual DM production. Santos et al. (2013) concluded that the supply of nutrient source (N source) in the growth medium enhances the forage yield of the tropical grass by direct influence on plant growth and development (tillers and leaves) and by promoting better plant root system.

### Effects of Wastewater on Plant Nutrient Uptake and Nutrient Budgeting

The results of the plant tissue analysis suggest that AWW-irrigated plants had higher nutrient uptake from the soil, based on higher tissue concentrations of N, P and K, and that the accumulation of primary nutrients (N-P-K) was higher under wastewater irrigation. The concentration of nutrients in the tissues of the plants in CI, CNI and CTRL soils varied significantly. The order of uptake found in the current study was as follows: AWW 150 > AWW 100 > TW 150 > TW 100 % FC. The nutrient uptake and DM yield potential were directly related to each other. For example, the CI soil with AWW 150 % FC showed higher DM yield and higher nutrient uptake (tissue concentration); similarly, CTRL soil with TW 100 % FC showed poor yield and nutrient uptake.

### Nutrient Budgeting for Effective Nutrient Management

Nutrient budgeting is an essential tool to measure and monitor the nutrient input and output ratio in a farmland (Oenema et al., 2003). This section shows the results obtained from the plant growth experiment and nutrient budgeting based on the nutrient loading through wastewater and discusses the differences between the predicted values and the measured value to evaluate the loss of nutrients as utilised by plants. Overall, the CI soils demonstrated good response in terms of input and output ratio as a result of high DM yield. In the CI soils, the nutrient budgeting showed that the applied (100 %) nutrients were utilised by the crop uptake (41 %) and soil storage (49.2 ± 5 % as measured) and very minimum was recorded as possible loss (less than 9.3 ± 7 % (as predicted) (Table 4).

**Table 4:** Nutrient budget for CI soils (*n* = 48)

Treatment types CI-Soils	Input soil (mg/pot)			Input (mg/pot) Water/wastewater			DM yield	Output (mg/pot)			Soil storage (predicted) (mg/pot)			Soil storage (measured) (mg/pot)		
	N	P	K	N	P	K	g/pot	N	P	K	N	P	K	N	P	K
NG (AWW 100 % FC)	3497.7	669.3	8549.4	2325	380	1250	163.2	5385.6 (3.3)	652.8 (0.4)	5712 (3.5)	437.1	396.5	4087.4	302.7	215.7	3530
ALF (AWW 100 % FC)	3497.7	669.3	8549.4	2325	380	1250	22	880 (4)	176 (0.8)	660 (3)	4942.7	873.3	9139.4	3416.7	714	5833.3
SF (AWW 100 % FC)	3497.7	669.3	8549.4	2325	380	1250	113.3	3965.5 (3.5)	793.1 (0.7)	5551.7 (4.9)	1857.2	256.2	4247.7	1823.2	220	3483.3
MUS (AWW 100 % FC)	3497.7	669.3	8549.4	2325	380	1250	102.3	3580.5 (3.5)	818.4 (0.8)	3478.2 (3.4)	2242.2	230.9	6321.2	1858.9	182	5607
NG (AWW 150 % FC)	3497.7	669.3	8549.4	4092	668.8	2200	171.6	5662.8 (3.3)	686.4 (0.4)	6006 (3.5)	1926.9	651.7	4788.4	1803.3	590.7	3729.3
ALF (AWW 150 % FC)	3497.7	669.3	8549.4	4092	668.8	2200	31.4	1413 (4.5)	251.2 (0.8)	942 (3)	6176.7	1086.9	9852.4	4070	799	8010
SF (AWW 150 % FC)	3497.7	669.3	8549.4	4092	668.8	2200	151.9	5924.1 (3.9)	1215.2 (0.8)	7443.1 (4.9)	1665.6	122.9	3351.3	1331.7	106	3133.3
MUS (AWW 150 % FC)	3497.7	669.3	8549.4	4092	668.8	2200	115	4025 (3.5)	920 (0.8)	4140 (3.6)	3564.7	418.1	6654.4	1886	383	6015
NG (TW 100 % FC)	3497.7	669.3	8549.4	0	0	0	39.9	1197 (3)	159.6 (0.4)	1396.5 (3.5)	2300.7	509.7	7197.9	1722.7	280.3	6333.3
ALF (TW 100 % FC)	3497.7	669.3	8549.4	0	0	0	11.7	421.2 (3.5)	81.9 (0.7)	386.1 (3.3)	3076.5	587.4	8208.3	2600	497	7733.3
SF (TW 100 % FC)	3497.7	669.3	8549.4	0	0	0	63.2	2212 (3.6)	442.4 (0.7)	2844 (4.5)	1285.7	226.9	5750.4	1206.7	167.3	5300
MUS (TW 100 % FC)	3497.7	669.3	8549.4	0	0	0	61.3	2206.8 (3.3)	490.4 (0.8)	2145.5 (3.5)	1290.9	178.9	6448.9	1111.7	108.7	6200
NG (TW 150 % FC)	3497.7	669.3	8549.4	0	0	0	51.8	1709.4 (3.3)	207.2 (0.4)	1813 (3.5)	1788.3	462.1	6781.4	1160	373.7	6670
ALF (TW 150 % FC)	3497.7	669.3	8549.4	0	0	0	16.7	567.8 (3.4)	130.2 (0.8)	501 (3)	2929.9	539.04	8093.4	2510	534.3	6415.7
SF (TW 150 % FC)	3497.7	669.3	8549.4	0	0	0	79.4	2779 (3.5)	555.8 (0.7)	3573 (4.5)	718.7	113.5	5021.4	612.7	101.7	5000.3
MUS (TW 150 % FC)	3497.7	669.3	8549.4	0	0	0	74.9	2621.5 (3.5)	599.2 (0.8)	2696.4 (3.6)	876.2	70.1	5898	812	50	5200

Values in the brackets are plant tissue concentration in % (3 kg soil per pot (934.4 mg/kg N; 188.4 mg/kg P; 2901 mg/kg K; AWW 186 mg/L N; 30.4 mg/L P; 100 mg/L K)

*NG* Napier grass, *ALF* alfalfa, *SF* sunflower, *MUS* mustard, *AWW* abattoir wastewater, *TW* tap water, *FC* field capacity

The CNI soils showed similar trends as CI soils with crop uptake (35.9 %), soil storage (54.7 %) and possible loss (9.4 %) (Table 5). In the case of CTRL soils, a completely different input-output ratio was observed since the soils’ original nutrient content was very low as compared to the CI and CNI. In this soil type, more than 47.2 % of the applied nutrients were utilised by the crop growth and development and 40.5 % was stored in the soil. The loss was 12.3 %. Similar estimates were observed by Hölscher et al. (1996) with a nutrient output of K (61 %) and P (62 %) (Table 6). Based on a study by Korom and Jeppson (1994) about 24 % of the N and 0.2 % of the TP applied leached through the soil zone of the sewer farm. Similarly, a study by Geber (2000) found that the amount of N removed increased with increased nutrient load. Applied amounts of P were the same as P in harvested biomass. In this experiment, three kinds of grass such as *Phalaris arundinacea* L., *Alopecurus pratensis* L. and *Bromus inermis Leyss* were irrigated with a mixture of treated effluent and supernatant at two levels of intensity. There were differences in DM yield between the grass species used in this experiment. Farm effluent is becoming increasingly recognised as an alternative source of irrigation water and should be utilised for its mineral content rather than waste disposal (Longhurst et al., 2000). It is best to develop environmentally friendly nutrient databases to guide producers in applying wastewater at correct N application rates that will result in better water quality and sustainable production systems (Kanwar et al., 2010).

**Table 5:** Nutrient budget for CNI soils (n = 48)

Treatment types CNI-Soils	Input Soil (mg/pot)			Input (mg/pot) Water/wastewater			DM yield	Output (mg/pot)			Soil storage (predicted) (mg/pot)			Soil storage (measured) (mg/pot)		
	N	P	K	N	P	K	g/pot	N	P	K	N	P	K	N	P	K
NG (AWW 100 % FC)	2803.2	565.2	8703	2325	380	1250	125	5385.6 (3.1)	652.8 (0.4)	5712 (3.6)	1253.2	445.2	5453.0	1074.0	410.0	5066.7
ALF (AWW 100 % FC)	2803.2	565.2	8703	2325	380	1250	30.7	880 (3.4)	176 (0.7)	660 (3.3)	4084.4	730.3	8939.9	1355.5	300	8000
SF (AWW 100 % FC)	2803.2	565.2	8703	2325	380	1250	95.7	3965.5 (3.5)	793.1 (0.7)	5551.7 (4.5)	1778.7	275.3	5646.5	1650.2	215.3	5166.7
MUS (AWW 100 % FC)	2803.2	565.2	8703	2325	380	1250	83.2	3580.5 (3.5)	818.4 (0.9)	3478.2 (3.6)	2216.2	196.4	6957.8	1355.3	162.7	6233.3
NG (AWW 150 % FC)	2803.2	565.2	8703	4092	668.8	2200	164.5	5662.8 (3.4)	686.4 (0.5)	6006 (3.6)	1302.2	411.5	4981.0	1050.0	340.7	4233.3
ALF (AWW 150 % FC)	2803.2	565.2	8703	4092	668.8	2200	36.4	1413 (4.4)	251.2 (0.8)	942 (3.2)	5293.6	942.8	9738.2	1693.6	413.0	8050.3
SF (AWW 150 % FC)	2803.2	565.2	8703	4092	668.8	2200	117.9	5924.1 (3.8)	1215.2 (0.7)	7443.1 (4.8)	2415.0	408.7	5243.8	2251.0	378.7	5033.3
MUS (AWW 150 % FC)	2803.2	565.2	8703	4092	668.8	2200	96.9	4025 (3.6)	920 (0.9)	4140 (3.7)	3406.8	361.9	7317.7	3180	297	7100
NG (TW 100 % FC)	2803.2	565.2	8703	0	0	0	29.5	1197 (3.3)	159.6 (0.6)	1396.5 (3.6)	1829.7	388.2	7641	431.0	320.0	7100.0
ALF (TW 100 % FC)	2803.2	565.2	8703	0	0	0	13.8	421.2 (3.8)	81.9 (0.8)	386.1 (3.6)	2278.8	454.8	8206.2	1050.0	209.0	8083.3
SF (TW 100 % FC)	2803.2	565.2	8703	0	0	0	48.6	2212 (3.3)	442.4 (0.8)	2844 (4.6)	1199.4	176.4	6467.4	1150.0	157.3	6050.0
MUS (TW 100 % FC)	2803.2	565.2	8703	0	0	0	48.4	2206.8 (3.6)	490.4 (0.9)	2145.5 (3.5)	1060.8	129.6	7009	1006.7	99.7	6533.3
NG (TW 150 % FC)	2803.2	565.2	8703	0	0	0	41.4	1709.4 (3.3)	207.2 (0.6)	1813 (3.5)	1437	316.8	7254	766	293.3333	7036.6667
ALF (TW 150 % FC)	2803.2	565.2	8703	0	0	0	17.2	567.8 (3.3)	130.2 (0.8)	501 (3.9)	2235.6	427.6	8032.2	1333.2	257	8000
SF (TW 150 % FC)	2803.2	565.2	8703	0	0	0	57.6	2779 (3.1)	555.8 (0.7)	3573 (4.5)	1017.6	162	6111	1000.333	151.3333	6066.6667
MUS (TW 150 % FC)	2803.2	565.2	8703	0	0	0	55.6	2621.5 (3.1)	599.2 (0.6)	2696.4 (3.6)	1079.6	231.6	6701.4	1060	207	6050

Values in the brackets are plant tissue concentration in % (3 kg soil per pot (934.4 mg/kg N; 188.4 mg/kg P; 2901 mg/kg K; AWW 186 mg/L N; 30.4 mg/L P; 100 mg/L K)

*NG* Napier grass, *ALF* alfalfa, *SF* sunflower, *MUS* mustard, *AWW* abattoir wastewater, *TW* tap water, *FC* field capacity

**Table 6:** Nutrient budget for CTRL soils (*n* = 48)

Treatment types CTRL-Soils	Input Soil (mg/pot)			Input (mg/pot) Water/wastewater			DM yield	Output (mg/pot)			Soil storage (predicted) (mg/pot)			Soil storage (measured) (mg/pot)		
	N	P	K	N	P	K	g/pot	N	P	K	N	P	K	N	P	K
NG (AWW 100 % FC)	1912.2	88.2	266.7	2325	380	1250	36.4	5385.6 (3)	652.8 (0.5)	5712 (3.5)	3145.2	286.2	242.7	1520.0	256.7	198.0
ALF (AWW 100 % FC)	1912.2	88.2	266.7	2325	380	1250	24	880 (3.1)	176 (0.6)	660 (3.3)	3493.2	324.2	724.7	1000.0	233.7	633.3
SF (AWW 100 % FC)	1912.2	88.2	266.7	2325	380	1250	70.6	3965.5 (3.3)	793.1 (0.6)	5551.7 (1.5)	1907.4	44.6	457.7	1506.7	35.4	408.3
MUS (AWW 100 % FC)	1912.2	88.2	266.7	2325	380	1250	55.4	3580.5 (3.5)	818.4 (0.8)	3478.2 (1.6)	2298.2	25	630.3	2080.0	22.0	600.7
NG (AWW 150 % FC)	1912.2	88.2	266.7	4092	668.8	2200	71.1	5662.8 (3.4)	686.4 (0.5)	6006 (2.6)	3586.8	401.5	618.1	3079.7	353.7	578.3
ALF (AWW 150 % FC)	1912.2	88.2	266.7	4092	668.8	2200	34.1	1413 (3.6)	251.2 (0.8)	942 (3.2)	4776.6	484.2	1375.5	2833.3	364.0	833.3
SF (AWW 150 % FC)	1912.2	88.2	266.7	4092	668.8	2200	99.5	5924.1 (3.8)	1215.2 (0.7)	7443.1 (1.8)	2223.2	60.5	675.7	2180.0	46.3	605.0
MUS (AWW 150 % FC)	1912.2	88.2	266.7	4092	668.8	2200	71	4025 (3.9)	920 (0.8)	4140 (1.7)	3235.2	189	1259.7	3000.0	92.0	1100.0
NG (TW 100 % FC)	1912.2	88.2	266.7	0	0	0	8.6	1197 (3.4)	159.6 (0.6)	1396.5 (1.6)	1619.8	36.6	129.1	900.0	29.3	93.7
ALF (TW 100 % FC)	1912.2	88.2	266.7	0	0	0	9.9	421.2 (3.9)	81.9 (0.8)	386.1 (1.8)	1526.1	9	88.5	862.0	4.7	64.7
SF (TW 100 % FC)	1912.2	88.2	266.7	0	0	0	31.5	2212 (3.6)	442.4 (0.2)	2844 (0.8)	778.2	25.2	14.7	520.0	17.3	10.0
MUS (TW 100 % FC)	1912.2	88.2	266.7	0	0	0	27.7	2206.8 (3.6)	490.4 (0.28)	2145.5 (0.9)	915	10.64	17.4	1000.0	7.4	9.0
NG (TW 150 % FC)	1912.2	88.2	266.7	0	0	0	14.7	1709.4 (3.3)	207.2 (0.5)	1813 (1.5)	1427.1	14.7	46.2	940.0	13.7	38.0
ALF (TW 150 % FC)	1912.2	88.2	266.7	0	0	0	12.2	567.8 (3.3)	130.2 (0.6)	501 (1.9)	1509.6	15	34.9	970.0	12.4	27.7
SF (TW 150 % FC)	1912.2	88.2	266.7	0	0	0	44.1	2779 (3.3)	555.8 (0.19)	3573 (0.5)	456.9	4.41	46.2	501.7	3.4	36.0
MUS (TW 150 % FC)	1912.2	88.2	266.7	0	0	0	32.7	2621.5 (3.2)	599.2 (0.18)	2696.4 (0.6)	865.8	29.34	70.5	836.7	15.5	60.0

Values in the brackets are plant tissue concentration in % (3 kg soil per pot (637.5 mg/kg N;29.4 mg/kg P; 88.9 mg/kg K; AWW 186 mg/L N; 30.4 mg/L P; 100 mg/L K)

*NG* Napier grass, *ALF* alfalfa, *SF* sunflower, *MUS* mustard, *AWW* abattoir wastewater, *TW* tap water, *FC* field capacity

## Conclusions

The overall DM yield was compared between the soils and the treatments. Under wastewater irrigation, among the four species grown in the CI soil, *P. purpureum* and *H. annuus* showed high biomass yields, followed by *S. alba* and *M. sativa*. The application of nutrient-rich wastewater increased biomass yield and plant height significantly in all the tested plants. Plants were grown under currently irrigated (CI) soil with 100 % field capacity showed significantly higher biomass yield than did those under the non-irrigated control field (CTRL) soil, which is attributed to higher nutrient input from AWW irrigation.

The plants grown under CTRL soil with TW irrigation showed significantly lower biomass yield. Similarly, the yield of CI and CNI soil were also found to be less in TW-irrigated soil due to poor nutrient supply. On the other hand, the application of AWW in the low fertile soil increased nutrient availability to plants thereby increasing plant height and DM yield. The plants grown under tap water showed about 70 % lower yields compared to the AWW irrigation. Similar trends in the biomass yields were observed for CNI and CTRL soils under the two water treatments, with the biomass yields in the following order: CI > CNI > CTRL soils. The results confirm the beneficial effects of AWW irrigation at the greenhouse level. However, a proper cropping pattern and wastewater irrigation management plan are essential to utilise the nutrients available in the wastewater-irrigated land treatment sites. The increase in fertility is evident from the effects of wastewater on biomass growth and also the abundance of nutrients accumulated in plants. A mass balance calculation on the applied, residual and the plant-accumulated nutrients over a few cropping periods will help us in understanding the nutrient cycling processes involved in the abattoir-irrigated land treatment sites, which will serve as an effective tool for the environmental management.
